# Neuro-immune-epithelial pathways involving substance P may contribute to mucosal pathology in gastro-oesophageal reflux disease

**DOI:** 10.3389/fimmu.2026.1743252

**Published:** 2026-04-27

**Authors:** Tom Leech, Philip Woodland, Madusha Peiris

**Affiliations:** Wingate Institute of Neurogastroenterology, Blizard Institute, The Faculty of Medicine and Dentistry, Queen Mary University of London, London, United Kingdom

**Keywords:** gastro-oesophageal reflux disease, inflammation, mast cells, neuro-immune, substance P

## Abstract

**Introduction:**

Substance P (SP) is contained in nerve fibres innervating the oesophageal mucosa and is released into the mucosa during oesophageal acid perfusion. However, the downstream effects of this peripheral SP are not known. We investigated the expression of SP receptors in the oesophageal mucosa of healthy controls and GORD patients, localisation of mast cells in relation to SP+ nerve fibres, and effects of SP exposure on oesophageal epithelial cells.

**Methods:**

Distal oesophageal biopsies were collected from healthy controls (HC; N = 10), functional heartburn (FH; N = 10), non-erosive reflux disease (NERD; N = 14), and erosive reflux disease (ERD; N = 13) patients. IHC was used to determine the cellular expression of the SP receptor, NK1R, on E-Cadherin+, CD3+, and tryptase+ cells. NE-1 oesophageal epithelial cells were incubated with SP to investigate inflammatory pathway activation. The proportion and density of mast cells expressing an SP receptor, MRGPRX2, was analysed, and the distance between SP+ nerve fibres and mast cells was determined.

**Results:**

NK1R was expressed on epithelial cells and, rarely, on CD3+ T cells. NK1R fluorescence intensity was significantly higher in NERD and ERD oesophageal mucosa compared with HCs. In NE-1 cells, exposure to SP induced NF-κB phosphorylation, ameliorated by NK1R inhibition, and release of IL-6 and IL-8. In NERD and ERD mucosa, the density of MRGPRX2+ mast cells was significantly higher than HCs. Across all subjects, 25 SP+ nerves were detected; 32% were located within 10 µm of a mast cell, and 84% within 50 µm.

**Conclusion:**

Expressions of SP receptors NK1R and MRGPRX2 are upregulated in NERD and ERD oesophageal mucosa, on epithelial cells and mast cells, respectively. Mast cells are also closely apposed to SP+ nerve fibres in the oesophageal mucosa. Furthermore, SP directly induced inflammatory pathway activation and release of cytokines in oesophageal epithelial cells *in vitro*. Communication between SP+ nerve fibres, epithelial cells, and mast cells may be of pathological significance in GORD.

## Introduction

Gastro-oesophageal reflux disease (GORD) is a condition characterised by the presence of heartburn symptoms, which can arise via activation of sensory nerve fibres located in the oesophageal mucosa. Firing of mucosal sensory nerve fibres can occur by several mechanisms, including sensitisation driven by acidic refluxate acting on TRPV1 channels, as well as soluble mediators produced by stimulated epithelial or immune cells (1). Activation of these sensory nerve fibres results in visceral pain experienced as heartburn. In non-erosive reflux disease (NERD) and erosive reflux disease (ERD) and, to a lesser extent, functional heartburn (FH), oesophageal mucosal inflammatory markers are upregulated compared with healthy controls (2, 3). However, neuro-immune-epithelial interactions in these patients have not been explored in detail.

Substance P (SP) is a neuropeptide contained in both spinal and vagal nerve fibres in rodents (4–7), both of which innervate the oesophageal mucosa of mammals (8–11). SP+ nerve fibres are present within the human oesophageal mucosa (12), and SP levels are higher in NERD subjects compared with healthy controls, positively correlating with the Gastrointestinal Symptom Rating Score (GSRS) acid reflux score (13, 14). Indeed, upregulation in the number of SP-containing neurones at barrier sites is an observation common to many chronic (micro)inflammatory conditions in the skin, colon, small intestine, and oesophagus (13, 15–19). In fact, in the oesophageal mucosa of the cat, luminal exposure with pH2 solution induces neuronal release of SP into the mucosal tissue and supernatant (20).

Release of SP from activated nerve fibres in the periphery can contribute to neurogenic inflammation by activating SP receptors at the site of insult. SP, once released from activated peripheral fibres, has pro-inflammatory effects in many cell types (21), including chemoattraction of lymphocytes (22), monocytes (23), and neutrophils (24), endothelial permeability (25, 26), mast cell degranulation and chemokine production (27, 28), and inflammatory mediator production and TLR upregulation in epithelial cells (29, 30).

Many of these peripheral inflammatory effects are mediated via NK1R agonism, the preferential receptor for SP (31). NK1R mRNA expression has been investigated in GORD oesophagus, with conflicting results (13, 32). One study found a higher expression of NK1R in NERD subjects compared with healthy controls (13), whereas another found NK1R expression to be higher only in ERD oesophageal mucosa, specifically in male subjects (32). However, neither study used pH monitoring to classify subjects; therefore, no study has yet investigated NK1R mRNA expression or protein localisation/expression level in well-phenotyped GORD subjects.

Another SP receptor, MRGPRX2, has also recently been shown to participate in disease pathophysiology of IBD and IBS (33, 34). Activation of MRGPRX2 in human mast cells, or in mouse mast cells, the ortholog Mrgprb2, induces IgE-independent degranulation (27, 35). Several studies have confirmed that SP is an endogenous ligand of mast cell-expressed MRGPRX2, resulting in degranulation (36, 37). MRGPRX2-positive mast cells are abundant in inflamed colonic mucosa of ulcerative colitis patients (34), whereas rectal mast cells isolated from IBS subjects exhibit greater degranulation in response to MRGPRX2 agonists than those from healthy controls (33). Upon degranulation, mast cells release stored mediators including histamine and tryptase, which subsequently activate mucosal sensory nerve fibres, induce epithelial inflammation, and lead to barrier dysfunction. MRGPRX2 mRNA has been detected in the human oesophagus (38), although no study to date has investigated its cellular localisation, or whether its expression is augmented in GORD.

We hypothesised that SP, released from sensory nerve fibres in the oesophageal mucosa, may contribute to chronic inflammation in GORD subjects. We aimed to characterise the expression of the SP receptors, NK1R and MRGPRX2, in the oesophageal mucosa of healthy controls and GORD subjects. Informed by this characterisation data, we examined the effect of SP exposure on oesophageal epithelial cells *in vitro*. Understanding of the expression of SP receptors in the oesophageal mucosa could reveal pathways which exacerbate mucosal inflammation and sensory nerve activation following luminal acid exposure.

## Methods

### Study subjects

All patients were prospectively recruited following informed consent and were required to have a clinical history of problematic heartburn requiring investigation. The criteria for inclusion are as follows: 1) aged between 18 and 70 years, 2) had symptoms of at least moderate heartburn more than three times per week, and 3) had a clinical referral for endoscopic examination for investigation of symptoms. Patients were excluded if they had 1) previous upper gastrointestinal surgery, 2) if they had severe upper gastrointestinal motility disorders, 3) if they were on coagulopathy or concurrent anticoagulant medication, 4) if they were pregnant, 5) if they were allergic or hypersensitive to local anaesthetic, and 6) if they had any other medical condition that would make it unsafe for the subject to participate, as determined by the treating physician.

Patients underwent endoscopy ± wireless ambulatory reflux monitoring. All patients had stopped PPI treatment for at least 7 days before endoscopy and reflux testing. Post-procedure, patients were divided according to phenotypes as follows: 1) erosive reflux disease (ERD), 2) nonerosive reflux disease (NERD), and 3) functional heartburn (FH) according to the definitions below. Patients with pathological acid exposure (>6% over the study period) on analysis of their reflux studies qualified for a diagnosis of NERD. Patients whose reflux testing studies did not meet pathological acid exposure criteria and had negative reflux/symptom association were classified as FH. Symptomatic patients with at least LA grade B esophagitis at endoscopy were classified as ERD. These phenotypic categorisations are supported by the Lyon Consensus 2.0 (39).

A total of 10 asymptomatic volunteers were included in this study. These volunteers had no history of gastrointestinal symptoms or anti-reflux medication us and had a Reflux Disease Questionnaire score of 0. Healthy controls were excluded if they 1) had previous upper GI surgery, 2) had severe upper GI motility disorders, 3) were pregnant, 4) were taking coagulopathy or concurrent anticoagulant medication, or 5) had any severe midface trauma or recent nasal surgery. All subjects had normal oesophageal appearance on endoscopy. Distal oesophageal biopsies of these HCs were prepared and analysed in an identical fashion to the patient biopsies used in this study.

A total of 32 GORD patients and 10 healthy controls included for immunohistochemical analysis in this study were recruited from the Royal London Hospital (Barts and the London School of Medicine and Dentistry, Queen Mary University of London, UK). This study was granted ethical approval by the NRES Committee London—Queen Square (Study reference: 19/LO/1506). Numbers and demographics of subjects analysed for each analysis are shown in **Table 1**.

**Table 1:** Demographics of the subjects analysed in this study.

Group	NK1R + E-cadherin (Immunohistochemistry)					
Phenotype	n	Female:male	Age (mean)	Age range	Mean RDQ	Mean AET%
HC	9	7:2	30	20-70	0	N/A
FH	8	4:4	40	20-72	42.6	3.0
NERD	13	7:6	46	27-69	31.2	13.6
ERD	10	3:7	47	22-73	32.3	N/A
MRGPRX2 + tryptase (immunohistochemistry)						
Phenotype	n	Female:male	Age (mean)	Age range	Mean RDQ	Mean AET%
HC	10	8:2	30	20-70	0	N/A
FH	7	4:3	39	20-72	41.8	3.0
NERD	10	6:4	46	27-69	32.5	14.5
ERD	11	3:8	47	22-73	30.2	N/A
TACR1 (qPCR)						
Phenotype	n	Female:male	Age (mean)	Age range	Mean RDQ	Mean AET%
HC	10	8:2	30	20-70	0	N/A
FH	10	5:5	38	20-72	45.4	3.2
NERD	14	8:6	46	27-69	29.6	13.5
ERD	13	4:9	46	22-73	28.7	N/A
MRGPRX2 (qPCR)						
Phenotype	n	Female:male	Age (mean)	Age range	Mean RDQ	Mean AET%
HC	9	7:2	31	20-70	0	N/A
FH	6	4:2	37	29-56	44.7	3.0
NERD	10	5:5	45	27-69	33.1	12.6
ERD	13	4:9	46	22-73	28.7	N/A

Patient demographics and disease parameters of the subjects used for different analyses in this study.

### Immunohistochemistry

Biopsies were immediately fixed in 4% PFA (Sigma-Aldrich, Cat. Number 158127) in phosphate-buffered saline for 2 h. Biopsies were washed three times in PBS and placed in 30% sucrose in PBS for 24 h at 4 °C. Fixed tissue was embedded in optimum cutting temperature compound (OCT compound; Sakura Tissue-Tek, Cat. Number 4583) and frozen at –20 °C. Serial 10-μm sections were cut and mounted on positively charged glass slides (Thermo Scientific, Cat. Number J1800AMNZ).

For immunohistochemistry (IHC), slides were washed with PBS for 5 min to rehydrate the sections, before being blocked with protein block (Protein Block Serum-Free Ready-to-use, Dako, Cat. Number X0909) for 1 h. Sections were incubated with primary antibody in PBS with 0.2% Triton X-100 for 16-18 h at 4°C. Antibodies used were the following: E-Cadherin (1:600 dilution; monoclonal mouse; Invitrogen, Cat. Number 13-1700), NK1R (1:200 dilution; polyclonal rabbit; Novus Biologicals, Cat. Number NB300-101), CD3 (1:100 dilution; monoclonal mouse; Dako, Cat. Number M7254), tryptase (1:300 dilution; monoclonal mouse; Thermo Fisher, Cat. Number MA5-11711), MRGPRX2 (1:200 dilution; polyclonal rabbit; Thermo Fisher, Cat. Number PA5-113198), Substance P (1:400 dilution; monoclonal rat; Santa Cruz Biotechnology, Cat. Number NC1/34HL). Slides were washed in PBS three times and incubated with 1:400-diluted secondary antibody (donkey anti-mouse 488 nm; donkey anti-mouse 568 nm; donkey anti-rat 488 nm; donkey anti-rabbit 568 nm; Invitrogen, Thermo Fisher Scientific; 1:400 dilution) at room temperature for 1 h. Slides were then washed in PBS three times and mounted with a coverslip with antifade mounting medium containing a DAPI fluorescent stain (Vector Laboratories, Cat. Number H-1500).

### NK1R fluorescence intensity quantification

Images were taken at 20× using an Olympus BX63 microscope and were captured using Olympus cellSens software. To reduce the risk of observer bias, all samples were anonymised prior to analysis. A minimum of three fields of view (FOVs) per sample were captured, and staining of NK1R was quantified. An AOI was drawn manually around the oesophageal epithelium, excluding papillae, and the mean staining intensity of the NK1R channel in the AOI was measured using ImageJ (U.S. National Institutes of Health, USA). The mean fluorescence intensity of an area adjacent to the tissue sample was quantified and subtracted from the NK1R signal, to minimise background interference.

### Analysis of gene expression in oesophageal biopsies

Endoscopic biopsies from the distal oesophagus were placed in RNAlater solution (Sigma, Cat. Number R0901-100ml) and stored at −80 °C. RNA was extracted using the RNeasy Mini Kit (Qiagen, Cat. Number 74016) according to the manufacturer’s instructions. Eluted RNA was quantified using NanoDrop (Thermo Scientific), using RNase-free water as a blank. Only samples with >40 ng/μl RNA and a 260/280 ratio >2.0 were used for qPCR experiments. RNA samples were diluted with RNase-free water such that 0.5 μg total RNA was reverse transcribed into cDNA using the QuantiTect reverse transcription kit (Qiagen, Cat. Number 205310).

qRT-PCR was performed on the AB7300 Real-Time PCR system (Applied Biosystems) using PowerUp SYBR Green Master Mix (Applied Biosystems; Cat. Number A25742). 25 ng of reverse-transcribed cDNA was used per 25-µL reaction. RT^2^ Primer Assays (Qiagen, Cat. Number 330001) were used at manufacturer-recommended concentrations. Relative gene expression by qPCR was calculated using the 2−ΔCt method, where gene expression was relative to the housekeeping gene.

### Substance P in vitro assay

NE-1 cells (AddexBio, Cat. Number T0013001) were cultured in keratinocyte serum-free media (KSFM; Gibco, Cat. Number 17005042) supplemented with penicillin/streptomycin (50 U/mL; Gibco, Cat. Number 15140122) in 5% CO_2_ at 37 °C. Cells between passages 4 and 10 were seeded at a concentration of 2×10^5^ cells/well into 12-well plates. Cells were incubated for 2 days, and media were changed each day prior to the assay.

On the day of the assay, wells were rinsed once with KSFM and incubated with KSFM, 30 nM, 100 nM, or 300 nM Substance P (Tocris, Cat. Number 1156). Cells were incubated for between 30 min and 24 h, depending on downstream analysis.

To determine the role of the NK1R receptor in NF-κB phosphorylation, a proportion of experiments utilised pretreatment with aprepitant, a specific irreversible NK1R antagonist (Cambridge Bioscience, Cat. Number T1743) (40, 41). Cells were treated with aprepitant at 0.1 or 1 µM for 30 min. Pretreatment solutions were removed, and Substance P at 300 nM was added for 30 min.

### Western blot

After incubation with Substance P for 30 min (NF-κB/p-NF-κB) or 90 min (NK1R), media were removed and wells rinsed once with ice-cold PBS. Next, 50 μL of ice-cold Laemmli buffer (5% v/v β-mercaptoethanol, 10% v/v glycerol, 0.02% v/v bromophenol blue, 2% v/v SDS, 0.0625M Tris–HCl pH 6.8) was added to each well. The plate was shaken for 15 min on ice before whole-cell lysate samples were collected and stored at −20°C.

Whole-cell lysate protein levels were quantified using The RC DC Protein Assay (Bio-Rad; Cat. Number 5000121), according to the manufacturer’s instructions. Samples were diluted to 0.5 mg/mL of protein using Laemmli buffer.

Samples were denatured at 95°C for 5 min, and 6 μg of sample was added per well to a 15-well 4%-20% polyacrylamide gel (Bio-Rad; Cat. Number 4561096).

Samples were run at 70 V for 15 min to allow the samples to enter the gel, and subsequently at 120 V until the samples had reached the bottom of the gel. Proteins were then transferred onto a nitrocellulose membrane at 300 mA for 90 min.

Membranes were blocked in 5% BSA in TBS-T (containing 0.1% Tween-20) for 1 h at room temperature and incubated at 4°C overnight in primary antibody. Primary antibodies used were NK1R (1:1,000 dilution; polyclonal rabbit; Novus Biologicals, Cat. Number NB300-101), NF-κB (1:1,000 dilution; monoclonal rabbit; Cell Signalling Technology, Cat. Number 8242), and p-NF-κB (1:1,000 dilution; monoclonal rabbit; Cell Signalling Technology, Cat. Number 3033).

Membranes were washed in TBS-T three times for 5 min and covered in 1:5,000-diluted HRP-conjugated secondary antibody solution (goat anti-mouse and goat anti-rabbit; Invitrogen, Thermo Fisher Scientific) for 90 min at room temperature. Membranes were washed in TBS-T 3 times for 5 min. Membranes were covered in approximately 1 mL of ECL™ Select Western Blotting Detection Reagent (Cytivia; Cat. Number GERPN2235), and chemiluminescence was detected using a ChemiDoc MP Imaging System (Bio-Rad; Cat. Number 12003154). Protein quantification data are presented relative to the housekeeping protein.

### Cytokine multiplex ELISA

To determine cytokine release, cells were incubated with KSFM alone or various concentrations of Substance P for 24 h. Supernatant was collected and analysed for the concentration of IL-8, IL-6, IL-1β, TNF-α, and NGF by custom ProcartaPlex multiplex ELISA (Thermo Fisher), according to the manufacturer’s instructions. Multiplex ELISA was analysed using a MAGPIX^®^ Instrument with xPONENT 4.3 software (Luminex). Samples were quantified using a 7-point standard curve.

### MRGPRX2+ mast cell quantification

MRGPRX2 was co-stained with mast cell tryptase, and double-positive cells were counted. For each sample, the total number of tryptase+ mast cells in an adjacent slide was quantified. Only mast cells in the epithelium and papillae were included in quantification. The area of the epithelium and papillae was determined and used to quantify the number of mast cells/mm^2^. These values were used to determine the proportion and density of MRGPRX2+ mast cells in the oesophageal mucosa of each subject.

### Distance of substance P+ nerve fibres to mast cells

Healthy control and GORD sample slides were stained with mast cell tryptase and Substance P. Mucosal Substance P+ nerve fibres in the epithelium and papillae were identified. When a Substance P+ nerve fibre was identified, the closest distance between a point on the nerve fibre and the nucleus of the closest tryptase+ mast cell was measured using the line tool of ImageJ.

### Statistical analysis

To compare results between healthy control and GORD phenotypes (FH, NERD, and ERD), all datasets were subject to the Shapiro–Wilk test for normality. If one or more groups failed the test for normal distribution, the Kruskal–Wallis test with Dunn’s *post-hoc* test was used to test for statistical significance between healthy controls and all GORD phenotypes. If all groups passed the Shapiro–Wilk normality test, one-way ANOVA with Dunnett’s *post-hoc* test was used to compare healthy controls with all GORD phenotypes.

For cell culture experiments, one-way ANOVA tests were used to analyse differences between groups. When ANOVA was positive, Tukey’s multiple comparisons test was used to identify which of the pairs was significantly different. All values are expressed as mean ± standard deviation (SD). All analysis and data visualisation was performed using GraphPad Prism Version 10 (GraphPad Software, LLC).

## Results

### Cellular expression of NK1R in the oesophageal mucosa of healthy controls and GORD subjects

**Figure 1:**
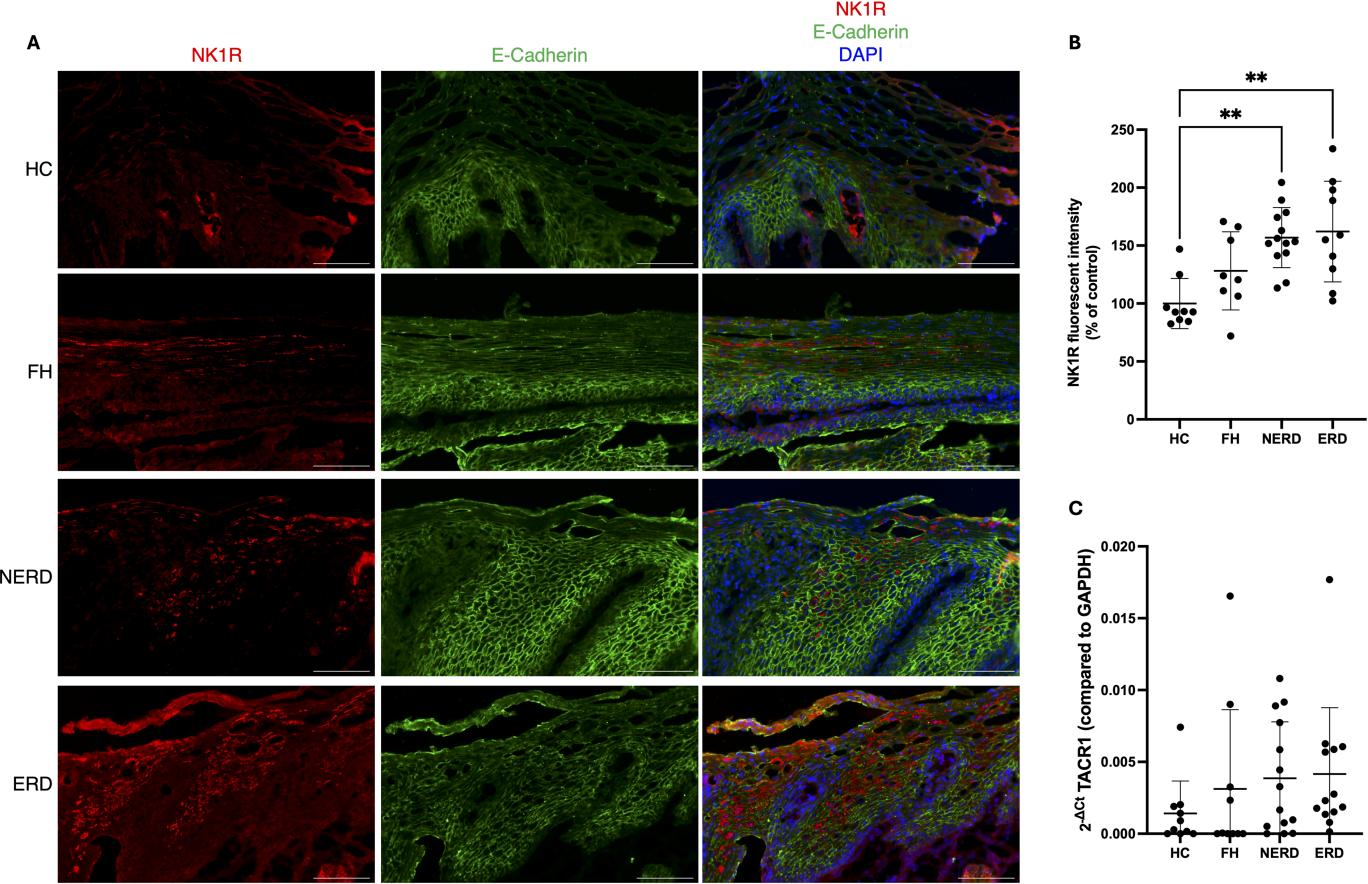
NK1R was expressed on epithelial cells in the oesophageal mucosa. **(A)** Representative images of NK1R expression in the oesophageal mucosa of healthy controls and GORD subjects, showing epithelial staining. **(B)** NK1R protein level, determined by fluorescence intensity, was higher in NERD and ERD subjects compared with healthy controls. **(C)** TACR1 mRNA was unchanged between healthy controls and any GORD group. Scale bar = 100 µm. Mean ± SD. ***p < 0.001. L = luminal side of the epithelium.

To determine potential functions of SP in the oesophageal mucosa, we investigated the expression of its receptor, NK1R, in biopsy samples of healthy controls and GORD subjects. We co-stained NK1R with E-cadherin to determine localisation of NK1R within the oesophageal epithelium and found NK1R predominantly expressed on E-cadherin-positive epithelial cells in both healthy and GORD patient biopsies (**Figure 1A**) with cytoplasmic staining detected across all subjects (**Supplementary Figure 1**). Quantification of NK1R staining showed elevated fluorescence intensity in the oesophageal epithelium of both NERD and ERD subjects compared with HCs (NERD = 1.57 fold-change, p = 0.0013; ERD = 1.62 fold-change, p = 0.0021; **Figure 1B**). There was also a trend towards higher NK1R fluorescence intensity in FH subjects, but this was not statistically significant (1.28 fold-change, p = 0.2678; **Figure 1B**).

Next, the mRNA expression level of TACR1—an NK1R-transcribing gene—was examined in healthy controls and GORD subjects by qPCR (HC = 10, FH = 10, NERD = 14, ERD = 13). In both HC and FH groups, TACR1 mRNA was detectable in 5/10 subjects; however, this increased in NERD and ERD to 11/14 and 13/13 subjects, respectively (**Figure 1C**). However, there was no significant difference in mean mRNA level between HCs and any GORD phenotype, possibly due to large intra-group variation (**Figure 1C**).

**Figure 2:**
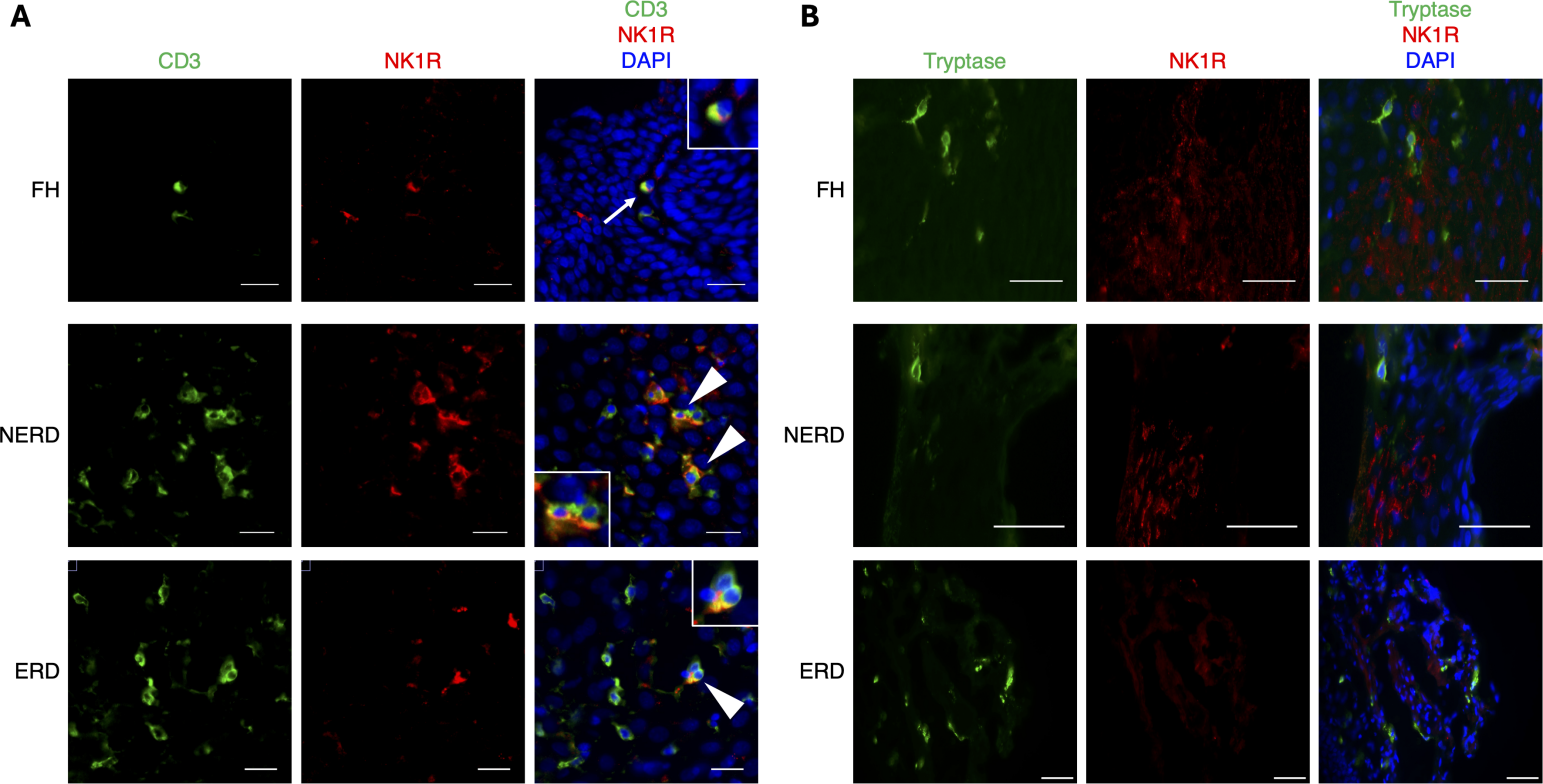
Expression of NK1R on T lymphocytes and mast cells in the oesophageal mucosa. **(A)** In the oesophageal mucosa, a small proportion of CD3+ T lymphocytes express NK1R. Of the 23 oesophageal mucosal biopsies analysed, 7 GORD samples, and no HC samples, contained NK1R+ CD3+ T cells. These cells were observed both intrapapillary (arrows) and within the epithelium (arrowheads). Scale bar = 20 µm. **(B)** In the oesophageal mucosa of healthy and GORD subjects, tryptase+ mast cells did not express NK1R. A total of 12 oesophageal biopsy samples were co-stained with tryptase and NK1R. Tryptase+ mast cells were observed in the epithelium and lamina propria. However, no double-labelled cells were detected in any sample. Scale bar = 50 µm.

NK1R expression was also detected on CD3+ T lymphocytes located in the oesophageal mucosa (**Figure 2A**), with NK1R+CD3+ cells detected in 7/23 samples studied (0/4 HC, 1/6 FH, 3/8 NERD, 3/5 ERD).

NK1R has also been detected on a human mast cell line (28), as well as tryptase+ mast cells in the skin (42). However, in a representative sample of healthy control and GORD mucosal biopsies (HC = 2, FH = 3, NERD = 3, ERD = 4), NK1R was not observed on tryptase+ mast cells (**Figure 2B**).

### Substance P induces NF-κB phosphorylation in cultured oesophageal epithelial cells

**Figure 3:**
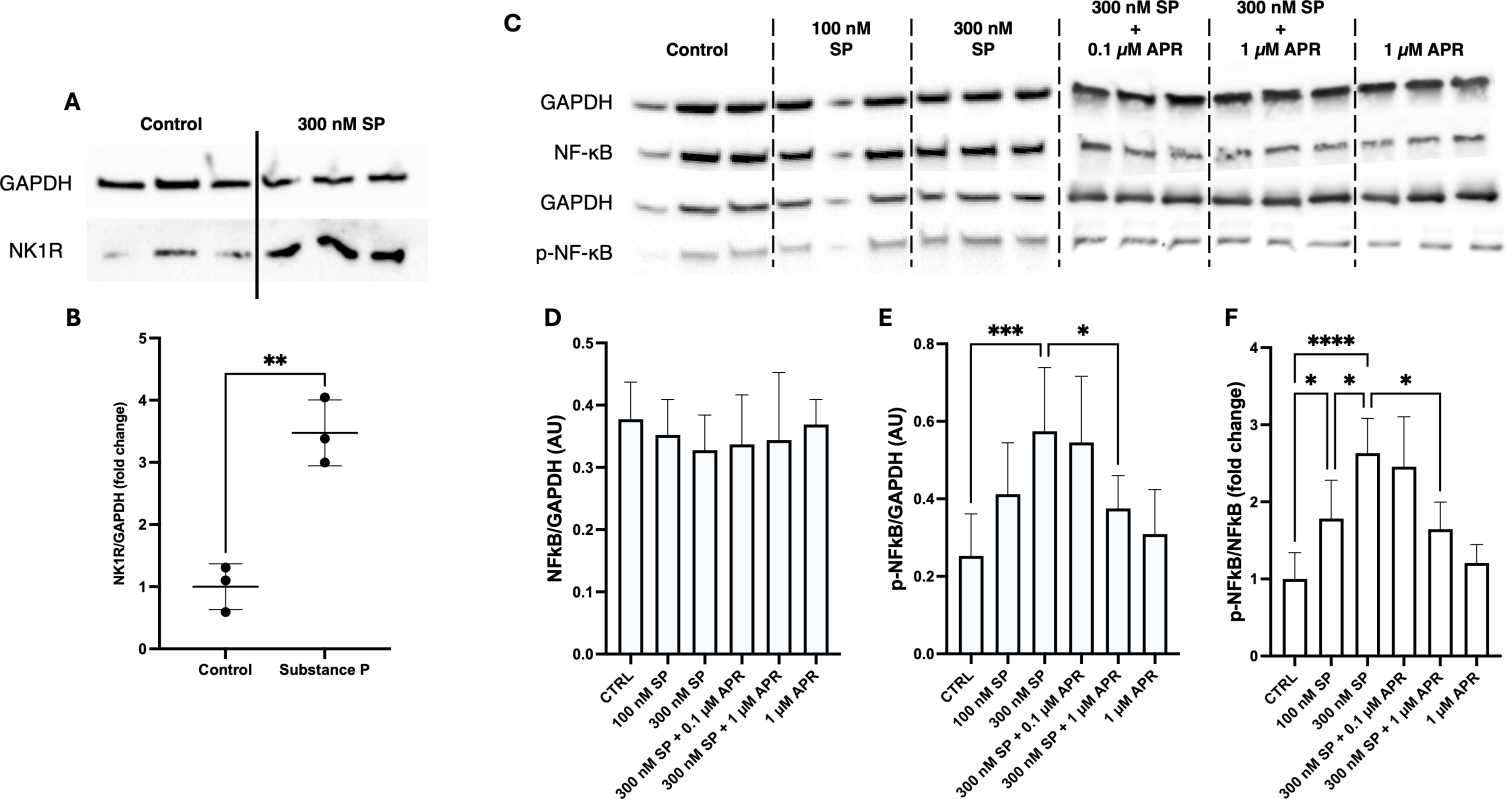
Substance P induces dose-dependent phosphorylation of NF-κB in NE-1 cells. After 30 min of incubation, both 100 and 300 nM substance P induced an increase in the p-NF-κB/NF-κB ratio in NE-1 cells (n=5), which is ameliorated by a 30-min pretreatment with aprepitant (n=3). **(A)** NK1R protein is detectable in unstimulated cells of the NE-1 cell line via Western blot, and **(B)** is significantly upregulated following 90 min of incubation with 300 nM Substance (P) Mean ± SD of three separate experiments. **(C)** Representative Western blot images showing protein levels of NF-κB and p-NF-κB in NE-1 cells. **(D)** Ratio of NF-κB to the loading control protein (GAPDH). **(E)** Ratio of p-NF-κB to the loading control protein (GAPDH). **(F)** Ratio of p-NF-κB/NF-κB protein levels. Mean ± SD. *p < 0.05, **p < 0.01, ***p < 0.001, ****p < 0.0001. SP, substance P; APR, aprepitant.

NK1R expression on oesophageal epithelial cells warranted investigating the effect of SP on cultured oesophageal epithelial cells (NE-1 cells) *in vitro*. First, we confirmed NK1R expression in the NE-1 oesophageal epithelial cell line using Western blot (**Figure 3A**). Furthermore, incubation with 300 nM SP induced further protein expression of NK1R, leading to a 3.5-fold greater protein level of NK1R after 90 min (p = 0.0027; **Figure 3B**).

To determine the effect of SP on inflammatory pathway activation, NE-1 cells were treated with media alone, 100 nM, or 300 nM SP for 30 min and the protein levels of total NF-κB and phosphorylated-NF-κB (p-NF-κB) were measured via Western blot. Incubation with 100 or 300 nM SP for 30 min did not affect the level of total NF-κB (p>0.05; **Figure 3D**). SP treatment for 30 min induced a dose-dependent increase to the p-NF-κB/NF-κB ratio of NE-1 cells (100 nM = 1.8 fold change, p = 0.035; 300 nM = 2.6 fold change, p < 0.0001; **Figure 2F**).

Pretreatment of NE-1 cells with the specific NK1R antagonist, 1 µM aprepitant, for 30 min significantly reduced the p-NF-κB/NF-κB ratio induced by 300 nM SP (2.6- vs. 1.6-fold, p = 0.014; **Figure 2F**), whereas pretreatment with 0.1 µM aprepitant did not significantly affect NF-κB phosphorylation compared with 300 nM SP (p = 0.99). Treatment with 1 µM aprepitant alone, without SP, also did not influence NF-κB phosphorylation compared with control (p = 0.99).

### Substance P induces inflammatory cytokine release in cultured oesophageal epithelial cells

**Figure 4:**
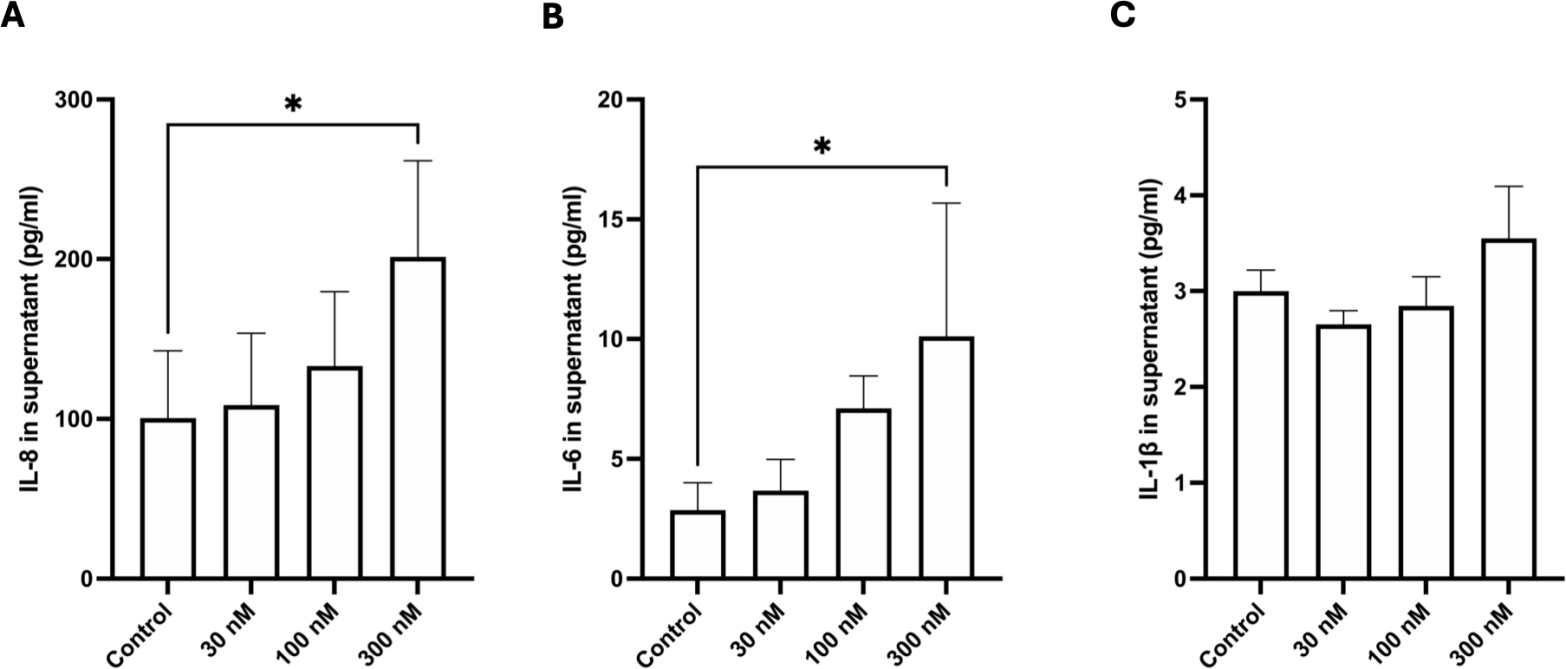
Substance P exposure induces dose-dependent release of IL-8 and IL-6 from NE-1 cells. After 24 h of incubation with KSFM media, 30, 100, or 300 nM Substance P, release of IL-8, IL-6, and IL1-β was determined by multiplex ELISA. **(a)** IL-8 concentration in the supernatant was higher than control in the 300-nM substance P group. **(b)** The concentration of IL-6 in the supernatant was higher than control in the 300-nM Substance P group, with a non-significant increase in the 100-nM substance P group. **(c)** The concentration of IL-1β in the media was not affected by 24-h incubation with any concentration of substance (P) Mean ± SD. *p < 0.05.

To determine whether activation of inflammatory signalling pathways results in measurable release of cytokines and other mediators from oesophageal epithelial cells, we incubated NE-1 cells with 30, 100, or 300 nM SP for 24 h and measured IL-8, IL-6, IL-1β, TNF-α, and NGF concentrations from cell supernatants. The mean concentration of IL-8 in the control samples was 100.6 pg/mL but was increased significantly in cells treated with 300 nM SP (201.4 pg/mL; p = 0.0373; **Figure 4A**). Similarly, cells treated with 300 nM SP (10.1 pg/mL; p = 0.0184; **Figure 4B**) had significantly higher IL-6 release compared with control samples (mean = 2.9 pg/mL). A non-significant trend towards higher IL-6 concentration was observed in 100-nM SP-treated samples (7.1 pg/mL; p = 0.1862). The IL-1β concentration did not differ between controls cells and those treated with SP at any concentration (**Figure 4C**). TNF-α and NGF protein levels were undetectable in all samples analysed.

### Oesophageal mucosal mast cells express MRGPRX2

**Figure 5:**
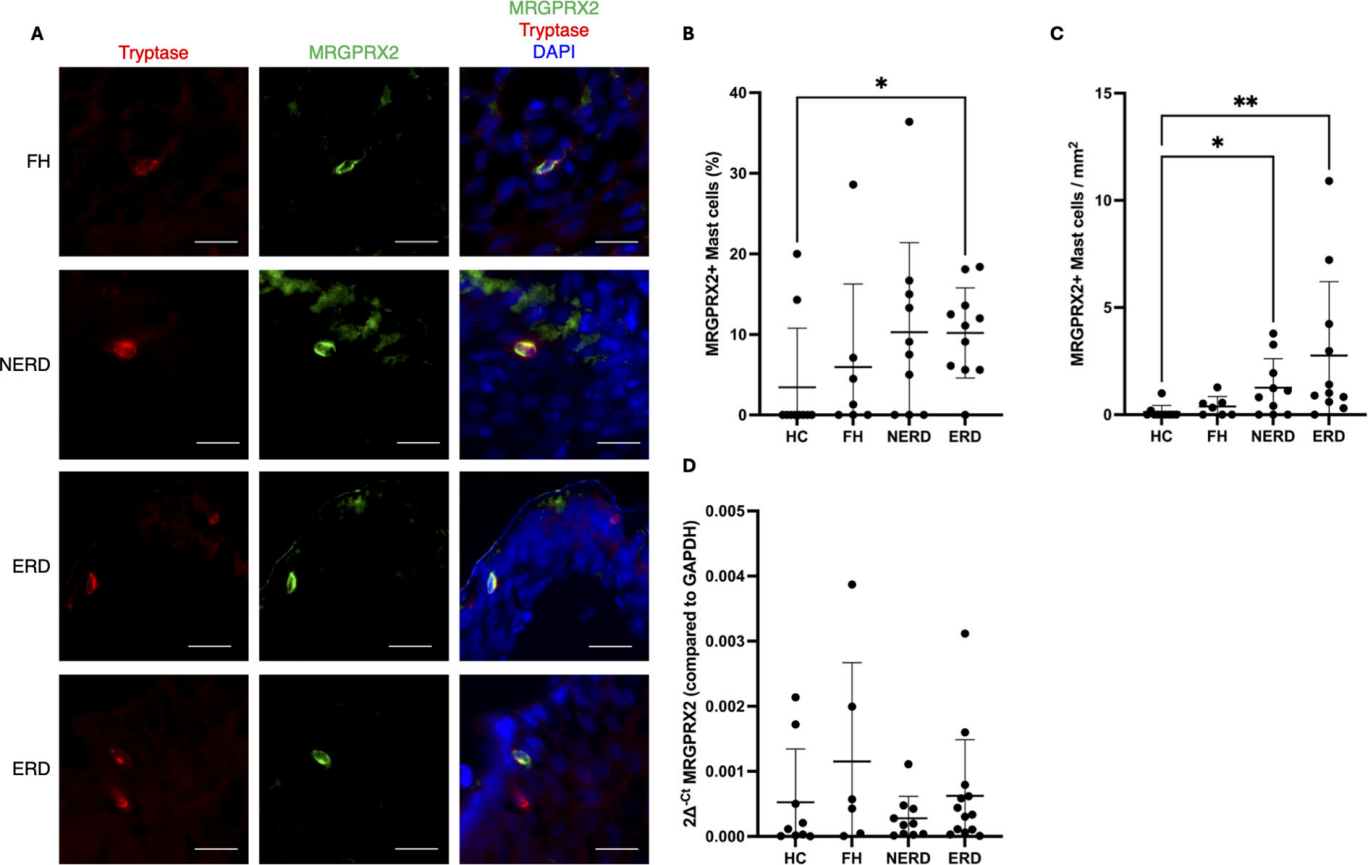
MRGPRX2+ mast cells are present in the oesophageal mucosa. **(A)** Representative images showing MRGPRX2 detected exclusively on tryptase-labelled mast cells in the oesophageal mucosa. **(B)** Quantification of the proportion of mast cells in the oesophageal epithelium, including papillae, that were MRGPRX2-positive. **(C)** Number of MRGPRX2+ mast cells/mm^2^ of oesophageal epithelium (including papillae). MRGPRX2-positive mast cells were rare in the healthy oesophageal mucosa but significantly more abundant in the mucosa of NERD and ERD subjects. **(D)** mRNA expression of MRGPRX2 in oesophageal mucosal samples. Scale bar = 10 µm. Mean ± SD. *p < 0.05, **p < 0.01.

MRGPRX2 was expressed exclusively at the cell membrane of tryptase+ mast cells in the oesophageal mucosa (**Figure 5A**). In HCs, 2/10 samples had at least one MRGPRX2-positive mast cell and an average of 3.4% MRGPRX2-positive mast cells (**Figure 5B**). In FH subjects, at least one MRGPRX2-positive mast cell was observed in 4/7 samples, although the overall proportion was not significantly different from HCs (5.9%, p > 0.05). A total of 7/10 NERD samples contained at least one MRGPRX2-positive mast cell, and there was a non-significant trend towards a higher proportion of MRGPRX2-positive mast cells in NERD subjects compared with HCs (10.3%, p = 0.18). However, 10/11 samples ERD subject biopsies had a minimum of one MRGPRX2-positive mast cell, and the proportion of MRGPRX2-positive mast cells was significantly higher than HCs (10.2%, p = 0.039).

The density of MRGPRX2+ mast cells/mm^2^ was also assessed, and it was found that the oesophageal epithelium (including papillae) of healthy controls contained an average of 0.12 MRGPRX2+ mast cells/mm^2^ (**Figure 5C**). MRGPRX2 density was not significantly different (0.38 MRGPRX2+ mast cells/mm^2^, p > 0.05) in FH subjects. However, in both NERD and ERD subjects, the number of MRGPRX2+ mast cells/mm^2^ was significantly higher than healthy controls (NERD = 1.26, p = 0.0428; ERD = 2.76, p = 0.0014). Interestingly, mRNA levels of MRGPRX2 were not significantly different between HCs and any GORD phenotype (p > 0.05; **Figure 5D**).

### Mast cells are closely apposed to substance P+ nerve fibres in the oesophageal mucosa

We analysed the distance between mucosal SP+ nerve fibres and the nucleus of its closest mast cell. A total of 25 nerve fibres were detected across all subjects (HC = 4, FH = 4, NERD = 6, ERD = 11). Whereas only 16% (4/25) of nerve fibres—across all subject groups—had a mast cell located within 5 μm, 32% (8/25) of nerve fibres had a mast cell within 10 μm, 56% (14/25) within 20 μm, and 84% (21/25) within 50 μm (**Table 2**).

**Table 2:** The distance from substance P+ nerve fibres and the closest mast cell in healthy and GORD subjects.

Closest mast cell to nerve fibre	HC	FH	NERD	ERD	Total
≤ 5 μm	0 (0.0%)	1 (25.0%)	1 (16.7%)	2 (18.2%)	4 (16.0%)
≤ 10 μm	1 (25.0%)	1 (25.0%)	1 (16.7%)	5 (45.5%)	8 (32.0%)
≤ 20 μm	1 (25.0%)	2 (50.0%)	4 (66.7%)	7 (63.6%)	14 (56.0%)
≤ 50 μm	3 (75.0%)	2 (50.0%)	6 (100.0%)	10 (90.9%)	21 (84.0%)
Number of nerve fibres observed	4	4	6	11	25

A total of 25 nerve fibres in the papillae and epithelium of the oesophagus were detected by substance P staining, and the distance to the nucleus of its closest mast cell is presented..

**Figure 6:**
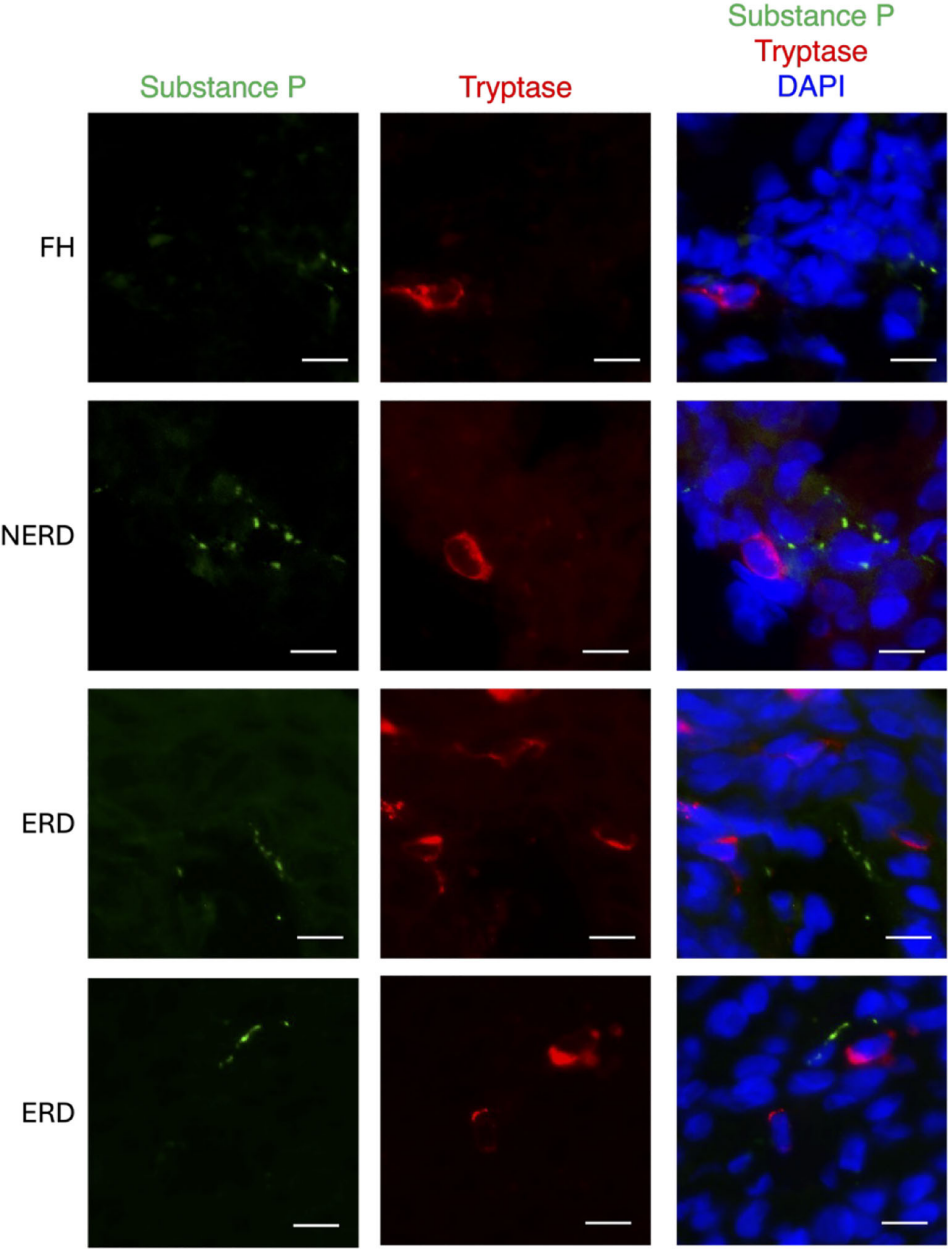
Representative images showing mast cells closely apposed to substance P+ nerve fibres in the oesophageal mucosa. Mucosal substance P+ nerve fibres were identified in the oesophageal epithelium and papillae. Many of these nerve fibres were in close apposition to tryptase+ mast cells. Scale bar = 10 µm. Adapted with permission from ‘Abstracts’ (2025) Neurogastroenterology & Motility, 37(S2). doi:10.1111/nmo.70126.

Representative images show close apposition of mast cells to SP+ nerve fibres in the oesophageal mucosa (**Figure 6**). SP is visible as characteristic punctate staining, whereas tryptase is visible as cytoplasmic staining within mast cells.

## Discussion

In this study, we illustrate a possible role of mucosal SP in promoting oesophageal epithelial inflammation and mast cell activation in GORD. We have demonstrated the expression of an SP receptor, NK1R, on T lymphocytes and epithelial cells in the oesophageal mucosa, as well as increased IHC fluorescence intensity of NK1R in the epithelium of both NERD and ERD subjects. Furthermore, we showed that exposure of oesophageal epithelial cells to nanomolar concentrations of SP *in vitro* results in an NF-κB-mediated inflammatory response via NK1R, leading to release of IL-8 and IL-6. We also demonstrated the expression of the SP receptor, MRGPRX2, on oesophageal mast cells, which were more frequently expressed in the mucosa of NERD and ERD subjects. Therefore, our data provide a foundation to support that SP, released by activated nerve fibres in the oesophageal mucosa, may interact with epithelial cells, T cells, and mast cells, to induce release of inflammatory mediators and mast cell degranulation.

NK1R expression in oesophageal mucosal biopsies has been previously investigated, where Kim et al. found NK1R mRNA expression to be significantly higher than healthy controls in ERD, but not NERD, subjects (32). In this study, NERD classification was defined by a positive PPI response rather than pH monitoring (32); however, a minority of FH patients respond to PPI treatment (43), and therefore, the NERD group may have included FH subjects, which could reduce the mean NK1R expression. Yoshida et al. found the NK1R mRNA expression to be significantly higher in NERD compared with healthy controls (13), but patient phenotyping using gold-standard oesophageal pH monitoring test was also lacking in this study. As such, our study is the first to investigate the expression of NK1R mRNA and protein in pH-phenotyped FH, NERD, and ERD subjects. Although healthy controls in our study did not undergo pH monitoring to confirm physiological reflux, all healthy controls had a reflux symptom score (RDQ) of 0, and normal oesophageal appearance at endoscopy.

NK1R protein has been detected in both human and rat keratinocytes (44, 45), human colonocytes (46), and isolated human nasal epithelial cells (30). Therefore, our finding of NK1R expression on human oesophageal epithelial cells is novel but in concordance with data at other barrier sites. Upregulation of this receptor in both NERD and ERD may contribute to increased activity of signalling downstream of NK1R activation by SP, such as NF-κB phosphorylation. Of note, the mRNA expression of the NK1R-coding gene, TACR1, did not significantly differ between healthy controls and any GORD group. This may be the result of posttranscriptional regulation driving differences in protein level. Although the specific pathway leading to increased NK1R expression in GORD patients is unclear, it is a finding noted in other conditions of chronic inflammation in both the skin and colon (44, 46). Indeed, the NK1R-coding gene contains a functional NF-κB binding site, which may contribute to its upregulation in inflammatory conditions (47). NK1R activation has also been demonstrated to induce its own expression (positive feedback) in several rodent cell types (48, 49), which is supported by our *in vitro* findings. This pathway may contribute to upregulated NK1R expression in NERD and ERD oesophageal mucosa.

In agreement with the vast majority of previous studies investigating the effects of SP on epithelial cells, we found a dose-dependent response in NF-κB phosphorylation after 30 min of incubation, accompanied by release of inflammatory mediators—and NF-κB targets—IL-8 and IL-6, over 24 h (29, 30, 50–54). For *in vitro* experimentation, we used high and low concentrations of SP (100/300 nM) based on previous studies demonstrating that incubation of various cell types, including GI tract epithelial cells, with SP in this range leads to NF-κB phosphorylation and subsequent immune mediator release (29, 30, 50–52). Our use of the specific NK1R, aprepitant (40), demonstrated the role of the NK1R receptor in driving SP-induced NF-κB phosphorylation in NE-1 cells. Therefore, NK1R identified in the human oesophageal epithelium *in situ* may contribute to SP-induced inflammatory signalling in these cells.

The release of IL-8 from epithelial cells has an established role in the recruitment of neutrophils, which results in epithelial permeability and mucosal inflammation (55, 56), whereas the functions of IL-6 at barrier sites are varied. In the skin and colon, IL-6 signalling is associated with tight junction remodelling, augmenting tissue repair, and promoting permeability (57–59). IL-6 signalling also activates the NF-κB pathway and has demonstrated inflammatory effects on many immune cell subtypes, as well as cultured intestinal epithelial cells (Caco-2) (60, 61). Interestingly, SP did not alter IL-1β secretion from NE-1 cells. Although the pro-IL-1β gene is a canonical target of NF-κB (62), the post-translational cleavage of pro-IL-1β into active IL-1β is tightly regulated (63). It is possible that, due to our specific *in vitro* approach, the second signal required for inflammasome formation, and subsequent pro-IL-1β cleavage and release, was not activated.

MRGPRX2 has received recent attention as a mast cell-specific SP receptor, which induces degranulation of human mast cells *in vitro* (27). Although MRGPRX2 mRNA has been identified in the oesophageal mucosa (38), our study is the first to investigate its protein expression as well as expression pattern in GORD patients. We confirmed that MRGPRX2 was exclusively expressed on mast cells in the oesophageal mucosa and that the proportion of MRGPRX2+ mast cells was greater in ERD than in healthy controls, with a non-significant trend in NERD subjects. Importantly, the density of MRGPRX2+ mast cells was greater in both NERD and ERD compared with healthy controls. Despite a higher density of MRGPRX2+ mast cells in NERD and ERD, MRGPRX2 mRNA did not differ significantly, which may be due to the overall scarcity of mast cells within the oesophageal mucosa. MRGPRX2+ mast cells appear to be dysregulated in inflammatory conditions of the intestine and skin, including IBS (33), ulcerative colitis (34), chronic prurigo (64), and rosacea (65), suggesting a role of this receptor in both visceral pain and inflammation via mast cell degranulation. However, the underlying cause of increased MRGPRX2 expression on mast cells at sites of inflammation is unclear.

Upon degranulation, mast cells release histamine, tryptase, and NGF, which are known to induce neuronal hypersensitivity and neurite outgrowth in rodent models and in human GI tract sensory nerves (66–71). Therefore, the proximity of mast cells to SP+ mucosal neurones illustrated in this study may be relevant to neuronal sensitisation and superficiality observed in GORD patients (72, 73). In NERD and ERD subjects, this elevation in MRGPRX2+ mast cells may contribute to inflammation and heartburn symptoms as a result of SP-induced degranulation, although further experimentation is required to determine functional significance.

Due to the paucity of nerve fibres in the oesophageal mucosa, we were limited to the identification of 25 nerve fibres across all healthy controls and GORD subjects, with 11 of these in separate ERD patients. Increased biopsy size or a greater number of sections analysed per sample may increase the number of identified SP+ nerve fibres. However, only two studies have previously illustrated the spatial relationship between mast cells and nerve fibres in the oesophagus, but only as a qualitative statement (74, 75). Therefore, we are the first to thoroughly analyse the proximity of mast cells to neurones in the oesophageal mucosa, with a sample of 25 total nerve fibres. This encourages further investigation into possible bidirectional methods of communication between these two cell types in the context of GORD. It is possible that SP, released by mucosal neurones upon stimulation by noxious refluxate or other mediators, induces degranulation of nearby MRGPRX2+ mast cells, which in turn further sensitise the neurones, as well as having effects at immune and epithelial cells. Further studies are required to validate this pathway, such as determining degranulation of oesophageal mast cells in response to MRGPRX2 agonists such as SP.

In conclusion, we have identified several potential molecular pathways which may be relevant to symptom generation in GORD. In general, interactions between inflammatory components and neurons leading to sensitisation and neuronal hyperactivity may contribute to PPI-refractory symptoms observed in up to 40% of GORD patients (76). Further studies are required to investigate whether these pathways are functional in the oesophageal mucosa *in situ*. However, by characterising the expression of SP receptors, and the inflammatory capacity of SP at oesophageal epithelial cells, we have demonstrated the potential importance of such neuro-immune-epithelial interactions in GORD.

## Data Availability

The raw data supporting the conclusions of this article will be made available by the authors, without undue reservation.

## Supplementary material

Supplementary Figure 1 NK1R was expressed in the cytoplasm of epithelial cells. In representative NERD and ERD samples, E-cadherin shows the membrane of oesophageal epithelial cells, and NK1R immunoreactivity is present within the cytoplasm. Scale bar = 10 µm.

## References

[1] Ustaoglu A Sawada A Lee C Lei WY Chen CL Hackett R . Heartburn sensation in nonerosive reflux disease: pattern of superficial sensory nerves expressing TRPV1 and epithelial cells expressing ASIC3 receptors. Am J Physiol Gastrointest Liver Physiol. (2021) 320:G804–G815. doi: 10.1152/ajpgi.00013.2021. PMID: 33655767

[2] Zavala-Solares MR Fonseca-Camarillo G Valdovinos M Granados J Grajales-Figueroa G Zamora-Nava L . Gene expression profiling of inflammatory cytokines in esophageal biopsies of different phenotypes of gastroesophageal reflux disease: a cross-sectional study. BMC Gastroenterol. (2021) 21:201. doi: 10.1186/s12876-021-01707-7. PMID: 33941087 PMC8094498

[3] Ergun P Kipcak S Gunel NS Bor S Sozmen EY . Roles of cytokines in pathological and physiological gastroesophageal reflux exposure. J Neurogastroenterol Motil. (2024) 30:290–302. doi: 10.5056/jnm22186. PMID: 37957115 PMC11238103

[4] Weissner W Winterson BJ Stuart-Tilley A Devor M Bove GM . Time course of substance P expression in dorsal root ganglia following complete spinal nerve transection. J Comp Neurol. (2006) 497:78–87. doi: 10.1002/cne.20981. PMID: 16680762 PMC2571959

[5] Smith GD Seckl JR Harmar AJ . Distribution of neuropeptides in dorsal root ganglia of the rat; substance P, somatostatin and calcitonin gene-related peptide. Neurosci Lett. (1993) 153:5–8. doi: 10.1016/0304-3940(93)90063-Q. PMID: 7685509

[6] Funakoshi K Kusakabe T Kadota T Goris RC Kishida R . Substance P immunoreactivity in the vagal nerve of mice. Neurosci Res. (1989) 7:235–48. doi: 10.1016/0168-0102(89)90018-7. PMID: 2482470

[7] Gamse R Lembeck F Cuello AC . Substance P in the vagus nerve. Naunyn-Schmiedeberg's Arch Pharmacol. (1979) 306:37–44. doi: 10.1007/BF00515591. PMID: 85263

[8] Neuhuber W Raab M Berthoud HR Wörl J . Innervation of the mammalian esophagus. In: Heidelberg: Springer. (2006). 16573241

[9] Dütsch M Eichhorn U Wörl J Wank M Berthoud HR Neuhuber WL . Vagal and spinal afferent innervation of the rat esophagus: A combined retrograde tracing and immunocytochemical study with special emphasis on calcium-binding proteins. J Comp Neurol. (1998) 398:289–307. doi: 10.1002/(SICI)1096-9861(19980824)398:2<289::AID-CNE9>3.0.CO;2-X 9700572

[10] Collman PI Tremblay L Diamant NE . The distribution of spinal and vagal sensory neurons that innervate the esophagus of the cat. Gastroenterology. (1992) 103:817–22. doi: 10.1016/0016-5085(92)90012-N. PMID: 1499932

[11] Khurana RK Petras JM . Sensory innervation of the canine esophagus, stomach, and duodenum. Am J Anat. (1991) 192:293–306. doi: 10.1002/aja.1001920309. PMID: 1759692

[12] Newton M Kamm MA Soediono PO Milner P Burnham WR Burnstock G . Oesophageal epithelial innervation in health and reflux oesophagitis. Gut. (1999) 44:317–22. doi: 10.1136/gut.44.3.317. PMID: 10026314 PMC1727420

[13] Yoshida N Kuroda M Suzuki T Kamada K Uchiyama K Handa O . Role of nociceptors/neuropeptides in the pathogenesis of visceral hypersensitivity of nonerosive reflux disease. Dig Dis Sci. (2013) 58:2237–43. doi: 10.1007/s10620-012-2337-7. PMID: 22899239

[14] Revicki DA Wood M Wiklund I Crawley J . Reliability and validity of the gastrointestinal symptom rating scale in patients with gastroesophageal reflux disease. Qual Life Res. (1997) 7:75–83. doi: 10.1023/A:1008841022998. PMID: 9481153

[15] Järvikallio A Harvima IT Naukkarinen A . Mast cells, nerves and neuropeptides in atopic dermatitis and nummular eczema. Arch Dermatol Res. (2003) 295:2–7. doi: 10.1007/s00403-002-0378-z. PMID: 12709813

[16] Amatya B El-Nour H Holst M Theodorsson E Nordlind K . Expression of tachykinins and their receptors in plaque psoriasis with pruritus. Br J Dermatol. (2011) 164:1023–9. doi: 10.1111/j.1365-2133.2011.10241.x. PMID: 21299544

[17] Patel M Valaiyaduppu Subas S Ghani MR Busa V Dardeir A Marudhai S . Role of substance P in the pathophysiology of inflammatory bowel disease and its correlation with the degree of inflammation. Cureus. (2020) 12:e11027. doi: 10.7759/cureus.11027. PMID: 33214955 PMC7671294

[18] Sohn W Lee OY Lee SP Lee KN Jun DW Lee HL . Mast cell number, substance P and vasoactive intestinal peptide in irritable bowel syndrome with diarrhea. Scand J Gastroenterol. (2013) 49:43–51. doi: 10.3109/00365521.2013.857712. PMID: 24256141

[19] Xu X Li Z Zou D Yang M Liu Z Wang X . High expression of calcitonin gene-related peptide and substance P in esophageal mucosa of patients with non-erosive reflux disease. Dig Dis Sci. (2013) 58:53–60. doi: 10.1007/s10620-012-2308-z. PMID: 22961239

[20] Harnett KM Rieder F Behar J Biancani P . Viewpoints on acid-induced inflammatory mediators in esophageal mucosa. J Neurogastroenterol Motil. (2010) 16:374–88. doi: 10.5056/jnm.2010.16.4.374. PMID: 21103419 PMC2978390

[21] O'Connor TM O'Connell J O'Brien DI Goode T Bredin CP Shanahan F . The role of substance P in inflammatory disease. J Cell Physiol. (2004) 201:167–80. doi: 10.1002/jcp.20061. PMID: 15334652

[22] Vishwanath R Mukherjee R . Substance P promotes lymphocyte-endothelial cell adhesion preferentially via LFA-1/ICAM-1 interactions. J Neuroimmunol. (1996) 71:163–71. doi: 10.1016/S0165-5728(96)00143-9. PMID: 8982116

[23] Ruff MR Wahl SM Pert CB . Substance P receptor-mediated chemotaxis of human monocytes. Peptides. (1985) 6 Suppl 2:107–11. doi: 10.1016/0196-9781(85)90142-1. PMID: 2417206

[24] Haines KA Kolasinski SL Cronstein BN Reibman J Gold LI Weissmann G . Chemoattraction of neutrophils by substance P and transforming growth factor-beta 1 is inadequately explained by current models of lipid remodeling. J Immunol. (1993) 151:1491–9. doi: 10.4049/jimmunol.151.3.1491 7687633

[25] Gao X Frakich N Filippini P Edwards LJ Vinkemeier U Gran B . Effects of substance P on human cerebral microvascular endothelial cell line hCMEC/D3 are mediated exclusively through a truncated NK-1 receptor and depend on cell confluence. Neuropeptides. (2022) 95:102265. doi: 10.1016/j.npep.2022.102265. PMID: 35696961

[26] Nguyen LS Villablanca AC Rutledge JC . Substance P increases microvascular permeability via nitric oxide-mediated convective pathways. Am J Physiol Regul Integr Comp Physiol. (1995) 268:R1060–R1068. doi: 10.1152/ajpregu.1995.268.4.R1060. PMID: 7537470

[27] Wollam J Solomon M Villescaz C Lanier M Evans S Bacon C . Inhibition of mast cell degranulation by novel small molecule MRGPRX2 antagonists. J Allergy Clin Immunol. (2024) 154:1033–43. doi: 10.1016/j.jaci.2024.07.002. PMID: 38971540

[28] Kulka M Sheen CH Tancowny BP Grammer LC Schleimer RP . Neuropeptides activate human mast cell degranulation and chemokine production. Immunology. (2008) 123:398–410. doi: 10.1111/j.1365-2567.2007.02705.x. PMID: 17922833 PMC2433325

[29] Koon HW Zhao D Zhan Y Simeonidis S Moyer MP Pothoulakis C . Substance P-stimulated interleukin-8 expression in human colonic epithelial cells involves protein kinase Cdelta activation. J Pharmacol Exp Ther. (2005) 314:1393–400. doi: 10.1124/jpet.105.088013. PMID: 15917399

[30] Larsson O Tengroth L Xu Y Uddman R Kumlien Georen S Cardell LO . Substance P represents a novel first-line defense mechanism in the nose. J Allergy Clin Immunol. (2018) 141:128–36. doi: 10.1016/j.jaci.2017.01.021. PMID: 28219705

[31] Regoli D Boudon A Fauchére JL . Receptors and antagonists for substance P and related peptides. Pharmacol Rev. (1994) 46:551–99. 7534932

[32] Kim JJ Kim N Park JH Kim YS Lee SM Lee DH . Comparison of tight junction protein-related gene mRNA expression levels between male and female gastroesophageal reflux disease patients. Gut Liver. (2018) 12:411–9. doi: 10.5009/gnl17419. PMID: 29558791 PMC6027836

[33] Decraecker L Cuende Estevez M Van Remoortel S Quan R Stakenborg N Wang Z . Characterisation of MRGPRX2(+) mast cells in irritable bowel syndrome. Gut. (2025) 74:1068–77. doi: 10.1136/gutjnl-2024-334037. PMID: 39988359

[34] Chen E Chuang LS Giri M Villaverde N Hsu NY Sabic K . Inflamed ulcerative colitis regions associated with MRGPRX2-mediated mast cell degranulation and cell activation modules, defining a new therapeutic target. Gastroenterology. (2021) 160:1709–24. doi: 10.1053/j.gastro.2020.12.076. PMID: 33421512 PMC8494017

[35] Van Remoortel S Lambeets L De Winter B Dong X Rodriguez Ruiz JP Kumar-Singh S . Mrgprb2-dependent mast cell activation plays a crucial role in acute colitis. Cell Mol Gastroenterol Hepatol. (2024) 18:101391. doi: 10.1016/j.jcmgh.2024.101391. PMID: 39179175 PMC11462171

[36] Raj S Hlushak S Arizmendi N Kovalenko A Kulka M . Substance P analogs devoid of key residues fail to activate human mast cells via MRGPRX2. Front Immunol. (2023) 14:1155740. doi: 10.3389/fimmu.2023.1155740. PMID: 37228611 PMC10203606

[37] Mousavizadeh R Waugh CM McCormack RG Cairns BE Scott A . MRGPRX2-mediated mast cell activation by substance P from overloaded human tenocytes induces inflammatory and degenerative responses in tendons. Sci Rep. (2024) 14:13540. doi: 10.1038/s41598-024-64222-1. PMID: 38866832 PMC11169467

[38] Porebski G Kwiecien K Pawica M Kwitniewski M . Mas-related G protein-coupled receptor-X2 (MRGPRX2) in drug hypersensitivity reactions. Front Immunol. (2018) 9:3027. doi: 10.3389/fimmu.2018.03027. PMID: 30619367 PMC6306423

[39] Gyawali CP Yadlapati R Fass R Katzka D Pandolfino J Savarino E . Updates to the modern diagnosis of GERD: Lyon consensus 2.0. Gut. (2024) 73:361–71. doi: 10.1136/gutjnl-2023-330616. PMID: 37734911 PMC10846564

[40] Tattersall FD Rycroft W Cumberbatch M Mason G Tye S Williamson DJ . The novel NK1 receptor antagonist MK-0869 (L-754,030) and its water soluble phosphoryl prodrug, L-758,298, inhibit acute and delayed cisplatin-induced emesis in ferrets. Neuropharmacology. (2000) 39:652–63. doi: 10.1016/S0028-3908(99)00172-0. PMID: 10728886

[41] Karthaus M Schiel X Ruhlmann CH Celio L . Neurokinin-1 receptor antagonists: review of their role for the prevention of chemotherapy-induced nausea and vomiting in adults. Expert Rev Clin Pharmacol. (2019) 12:661–80. doi: 10.1080/17512433.2019.1621162. PMID: 31194593

[42] Zhan M Zheng W Jiang Q Zhao Z Wang Z Wang J . Upregulated expression of substance P (SP) and NK1R in eczema and SP-induced mast cell accumulation. Cell Biol Toxicol. (2017) 33:389–405. doi: 10.1007/s10565-016-9379-0. PMID: 28154998

[43] Wong MW Yi CH Liu TT Lei WY Hung JS Wang JH . Mucosal integrity and acid sensitivity predict proton pump inhibitor response in patients with heartburn and normal acid exposure. Dig Liver Dis. (2025) 57:842–8. doi: 10.1016/j.dld.2025.01.182. PMID: 39864982

[44] Ohanyan T Schoepke N Eirefelt S Hoey G Koopmann W Hawro T . Role of substance P and its receptor neurokinin 1 in chronic prurigo: a randomized, proof-of-concept, controlled trial with topical aprepitant. Acta Derm Venereol. (2018) 98:26–31. doi: 10.2340/00015555-2780. PMID: 28853492

[45] Wei T Guo TZ Li WW Hou S Kingery WS Clark JD . Keratinocyte expression of inflammatory mediators plays a crucial role in substance P-induced acute and chronic pain. J Neuroinflammation. (2012) 9:181. doi: 10.1186/1742-2094-9-181. PMID: 22824437 PMC3458986

[46] Goode T Connell J Anton P Wong H Reeve J Sullivan GC . Neurokinin-1 receptor expression in inflammatory bowel disease: molecular quantitation and localisation. Gut. (2000) 47:387. doi: 10.1136/gut.47.3.387. PMID: 10940277 PMC1728039

[47] Simeonidis S Castagliuolo I Pan A Liu J Wang CC Mykoniatis A . Regulation of the NK-1 receptor gene expression in human macrophage cells via an NF-kappa B site on its promoter. Proc Natl Acad Sci USA. (2003) 100:2957–62. doi: 10.1073/pnas.0530112100. PMID: 12594338 PMC151448

[48] Koh YH Moochhala S Bhatia M . Activation of neurokinin-1 receptors up-regulates substance P and neurokinin-1 receptor expression in murine pancreatic acinar cells. J Cell Mol Med. (2012) 16:1582–92. doi: 10.1111/j.1582-4934.2011.01475.x. PMID: 22040127 PMC3823226

[49] Shi X Wang L Clark JD Kingery WS . Keratinocytes express cytokines and nerve growth factor in response to neuropeptide activation of the ERK1/2 and JNK MAPK transcription pathways. Regul Pept. (2013) 186:92–103. doi: 10.1016/j.regpep.2013.08.001. PMID: 23958840 PMC3799830

[50] Lieb K Fiebich BL Berger M Bauer J Schulze-Osthoff K . The neuropeptide substance P activates transcription factor NF-kappa B and kappa B-dependent gene expression in human astrocytoma cells. J Immunol. (1997) 159:4952–8. 9366421

[51] Zhao D Kuhnt-Moore S Zeng H Pan A Wu JS Simeonidis S . Substance P-stimulated interleukin-8 expression in human colonic epithelial cells involves Rho family small GTPases. Biochem J. (2002) 368:665–72. doi: 10.1042/bj20020950. PMID: 12169092 PMC1222994

[52] Li M Zhong X Xu WT . Substance P promotes the progression of bronchial asthma through activating the PI3K/AKT/NF-kappaB pathway mediated cellular inflammation and pyroptotic cell death in bronchial epithelial cells. Cell Cycle. (2022) 21:2179–91. doi: 10.1080/15384101.2022.2092166. PMID: 35726575 PMC9519020

[53] Sun J Ramnath RD Zhi L Tamizhselvi R Bhatia M . Substance P enhances NF-κB transactivation and chemokine response in murine macrophages via ERK1/2 and p38 MAPK signaling pathways. Am J Physiol Cell Physiol. (2008) 294:C1586–C1596. doi: 10.1152/ajpcell.00129.2008. PMID: 18434625

[54] Lai JP Lai S Tuluc F Tansky MF Kilpatrick LE Leeman SE . Differences in the length of the carboxyl terminus mediate functional properties of neurokinin-1 receptor. Proc Natl Acad Sci USA. (2008) 105:12605–10. doi: 10.1073/pnas.0806632105. PMID: 18713853 PMC2518097

[55] Souza RF Huo X Mittal V Schuler CM Carmack SW Zhang HY . Gastroesophageal reflux might cause esophagitis through a cytokine-mediated mechanism rather than caustic acid injury. Gastroenterology. (2009) 137:1776–84. doi: 10.1053/j.gastro.2009.07.055. PMID: 19660463

[56] Yamaguchi T Yoshida N Tomatsuri N Takayama R Katada K Takagi T . Cytokine-induced neutrophil accumulation in the pathogenesis of acute reflux esophagitis in rats. Int J Mol Med. (2005) 16:71–7. doi: 10.3892/ijmm.16.1.71 15942680

[57] Suzuki T Yoshinaga N Tanabe S . Interleukin-6 (IL-6) regulates claudin-2 expression and tight junction permeability in intestinal epithelium. J Biol Chem. (2011) 286:31263–71. doi: 10.1074/jbc.M111.238147. PMID: 21771795 PMC3173073

[58] Wang L Srinivasan S Theiss AL Merlin D Sitaraman SV . Interleukin-6 induces keratin expression in intestinal epithelial cells: potential role of keratin-8 in interleukin-6-induced barrier function alterations. J Biol Chem. (2007) 282:8219–27. doi: 10.1074/jbc.M604068200. PMID: 17213200

[59] Wang XP Schunck M Kallen KJ Neumann C Trautwein C Rose-John S . The interleukin-6 cytokine system regulates epidermal permeability barrier homeostasis. J Invest Dermatol. (2004) 123:124–31. doi: 10.1111/j.0022-202X.2004.22736.x. PMID: 15191552

[60] Tanaka T Narazaki M Kishimoto T . IL-6 in inflammation, immunity, and disease. Cold Spring Harb Perspect Biol. (2014) 6:a016295. doi: 10.1101/cshperspect.a016295. PMID: 25190079 PMC4176007

[61] Wang L Walia B Evans J Gewirtz AT Merlin D Sitaraman SV . IL-6 induces NF-κB activation in the intestinal epithelia. J Immunol. (2003) 171:3194–201. doi: 10.4049/jimmunol.171.6.3194. PMID: 12960348

[62] Cogswell JP Godlevski MM Wisely GB Clay WC Leesnitzer LM Ways JP . NF-kappa B regulates IL-1 beta transcription through a consensus NF-kappa B binding site and a nonconsensus CRE-like site. J Immunol. (1994) 153:712–23. 8021507

[63] Lopez-Castejon G Brough D . Understanding the mechanism of IL-1β secretion. Cytokine Growth Factor Rev. (2011) 22:189–95. doi: 10.1016/j.cytogfr.2011.10.001. PMID: 22019906 PMC3714593

[64] Kolkhir P Pyatilova P Ashry T Jiao Q Abad-Perez AT Altrichter S . Mast cells, cortistatin, and its receptor, MRGPRX2, are linked to the pathogenesis of chronic prurigo. J Allergy Clin Immunol. (2022) 149:1998–2009.e5. doi: 10.1016/j.jaci.2022.02.021. PMID: 35283140

[65] Roy S Alkanfari I Chaki S Ali H . Role of MrgprB2 in rosacea-like inflammation in mice: modulation by β-arrestin 2. J Invest Dermatol. (2022) 142:2988–2997.e3. doi: 10.1016/j.jid.2022.05.005. PMID: 35644498 PMC9634617

[66] Wouters MM Balemans D Van Wanrooy S Dooley J Cibert-Goton V Alpizar YA . Histamine receptor H1-mediated sensitization of TRPV1 mediates visceral hypersensitivity and symptoms in patients with irritable bowel syndrome. Gastroenterology. (2016) 150:875–887.e9. doi: 10.1053/j.gastro.2015.12.034. PMID: 26752109

[67] Amadesi S Nie J Vergnolle N Cottrell GS Grady EF Trevisani M . Protease-activated receptor 2 sensitizes the capsaicin receptor transient receptor potential vanilloid receptor 1 to induce hyperalgesia. J Neurosci. (2004) 24:4300–12. doi: 10.1523/JNEUROSCI.5679-03.2004. PMID: 15128844 PMC6729438

[68] Yu S Gao G Peterson BZ Ouyang A . TRPA1 in mast cell activation-induced long-lasting mechanical hypersensitivity of vagal afferent C-fibers in guinea pig esophagus. Am J Physiol Gastrointest Liver Physiol. (2009) 297:G34–G42. doi: 10.1152/ajpgi.00068.2009. PMID: 19423751

[69] Hasler WL Grabauskas G Singh P Owyang C . Mast cell mediation of visceral sensation and permeability in irritable bowel syndrome. Neurogastroenterol Motil. (2022) 34:e14339. doi: 10.1111/nmo.14339. PMID: 35315179 PMC9286860

[70] Zhang S Grabauskas G Wu X Joo MK Heldsinger A Song I . Role of prostaglandin D2 in mast cell activation-induced sensitization of esophageal vagal afferents. Am J Physiol Gastrointest Liver Physiol. (2013) 304:G908–G916. doi: 10.1152/ajpgi.00448.2012. PMID: 23471341 PMC3652067

[71] Dothel G Barbaro MR Boudin H Vasina V Cremon C Gargano L . Nerve fiber outgrowth is increased in the intestinal mucosa of patients with irritable bowel syndrome. Gastroenterology. (2015) 148:1002–1011.e4. doi: 10.1053/j.gastro.2015.01.042. PMID: 25655556

[72] Woodland P Shen Ooi JL Grassi F Nikaki K Lee C Evans JA . Superficial esophageal mucosal afferent nerves may contribute to reflux hypersensitivity in nonerosive reflux disease. Gastroenterology. (2017) 153:1230–9. doi: 10.1053/j.gastro.2017.07.017. PMID: 28734832

[73] Miwa H Kondo T Oshima T Fukui H Tomita T Watari J . Esophageal sensation and esophageal hypersensitivity - overview from bench to bedside. J Neurogastroenterol Motil. (2010) 16:353–62. doi: 10.5056/jnm.2010.16.4.353. PMID: 21103417 PMC2978388

[74] Ustaoglu A Daudali FA D'Afflitto M Murtough S Lee C Moreno E . Identification of novel immune cell signature in gastroesophageal reflux disease: altered mucosal mast cells and dendritic cell profile. Front Immunol. (2023) 14:1282577. doi: 10.3389/fimmu.2023.1282577. PMID: 38098488 PMC10720318

[75] Zhang S Shoda T Aceves SS Arva NC Chehade M Collins MH . Mast cell-pain connection in eosinophilic esophagitis. Allergy. (2022) 77:1895–9. doi: 10.1111/all.15260. PMID: 35175645 PMC9167217

[76] Rettura F Bronzini F Campigotto M Lambiase C Pancetti A Berti G . Refractory gastroesophageal reflux disease: a management update. Front Med (Lausanne). (2021) 8:765061. doi: 10.3389/fmed.2021.765061. PMID: 34790683 PMC8591082

