# Supplementary figures and images for: Neuro-immune-epithelial pathways involving substance P may contribute to mucosal pathology in gastro-oesophageal reflux disease
Source: Front Immunol. 2026 Apr 27;17:1743252. doi: 10.3389/fimmu.2026.1743252 (PMC13158196; doi:10.3389/fimmu.2026.1743252)

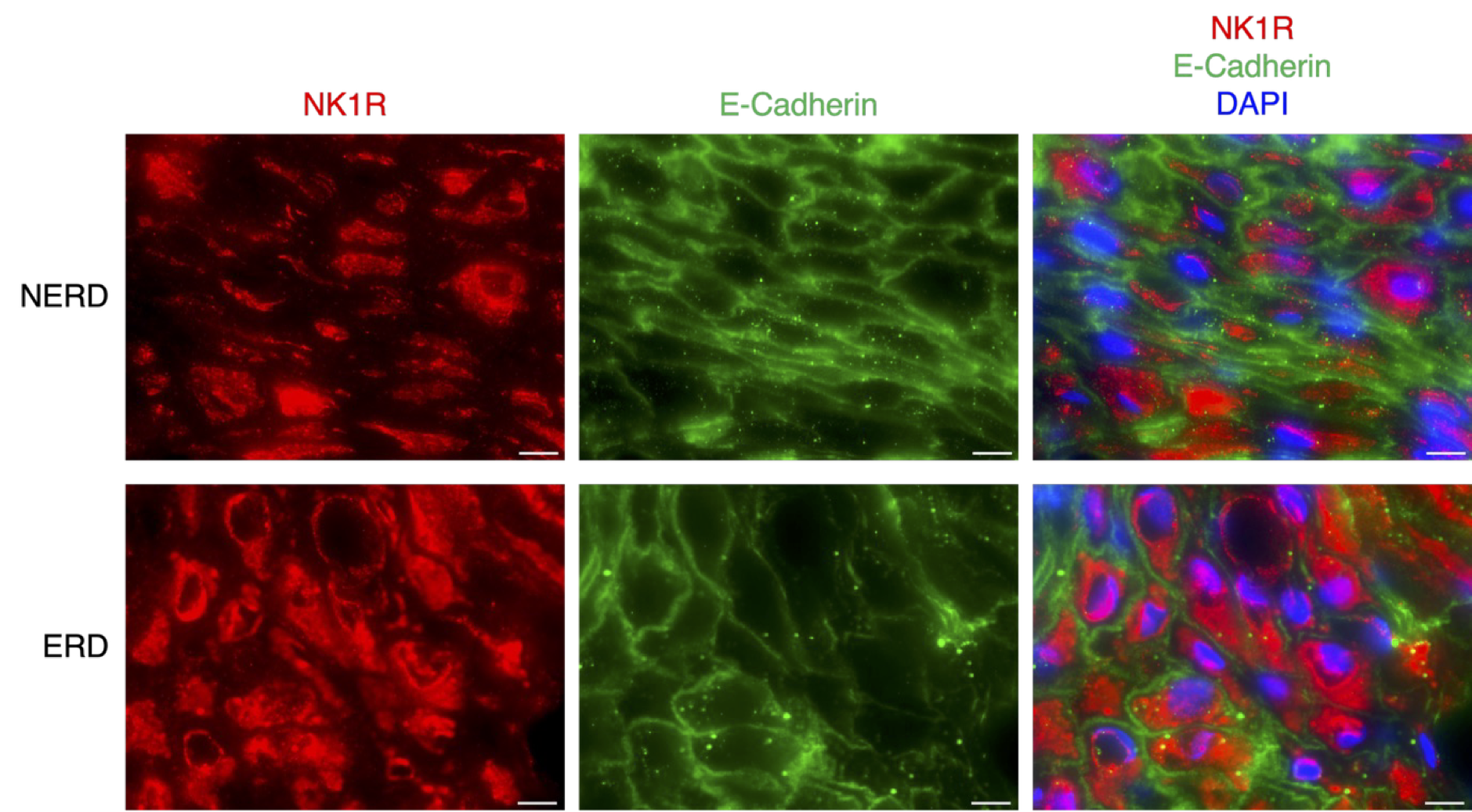

Supplement: Supplementary Figure 1 — NK1R was expressed in the cytoplasm of epithelial cells. In representative NERD and ERD samples, E-cadherin shows the membrane of oesophageal epithelial cells, and NK1R immunoreactivity is present within the cytoplasm. Scale bar = 10 µm. [file Image1.tiff]
